# Efficacy and safety of traditional Chinese medicine in managing bone loss post-endocrine therapy in hormone receptor-positive breast cancer patients

**DOI:** 10.1097/MD.0000000000039961

**Published:** 2024-10-11

**Authors:** Liuxiang Chen, Yuanqi Zhang, Jianwen Li, Attila Kalmar

**Affiliations:** a Center for Thyroid, Breast and Vascular Surgery, Affiliated Hospital of Guangdong Medical University, Zhanjiang, China; b Guangdong Medical University, Zhanjiang, China.

**Keywords:** bone density, endocrine therapy, hormone receptor-positive breast cancer, osteoporosis management, traditional Chinese medicine

## Abstract

**Background:**

This study aims to evaluate the efficacy and safety of traditional Chinese medicine (TCM) kidney-tonifying methods in treating bone loss and osteoporosis following endocrine therapy in patients with hormone receptor-positive breast cancer.

**Methods:**

This research systematically searched 6 major medical electronic databases, initially identifying 986 articles, ultimately including 22 randomized controlled trials. These studies encompassed a total of 1579 patients and investigated the impact of TCM kidney-tonifying methods on lumbar spine bone density, lumbar spine bone density *T*-values, femoral neck bone density, clinical efficacy, and drug safety. The Cochrane quality scoring system was utilized to assess the quality of the literature, and data were synthesized using meta-analysis techniques.

**Results:**

TCM kidney-tonifying methods significantly improved lumbar spine bone density (standardized mean difference [SMD] = 0.70, 95% confidence interval [CI]: 0.43–0.97) and lumbar spine bone density *T*-values (SMD = 1.012, 95% CI: 0.506–1.517). There was also a positive trend in enhancing femoral neck bone density (SMD = 0.645, 95% CI: 0.321–0.970). Although improvements in clinical efficacy did not reach statistical significance (relative risk [RR] = 1.122, 95% CI: 0.933–1.348), the studies indicated good safety of the treatment, with most studies reporting no significant adverse reactions.

**Conclusion:**

TCM kidney-tonifying methods may have a positive therapeutic effect on bone loss and osteoporosis following endocrine therapy in patients with hormone receptor-positive breast cancer, demonstrating good tolerability and safety. Given the current evidence, it is recommended to include TCM kidney-tonifying methods as a complementary therapeutic approach in treatment protocols. Future research should further validate these findings and explore their application in different patient subgroups.

## 1. Background

Breast cancer is the most common malignant tumor among women, with approximately 75% of patients presenting as hormone receptor positive, including estrogen receptors (ER) and/or progesterone receptors (PR).^[1]^ High expression of hormone receptors significantly increases the risk of distant metastases, adversely affecting prognosis.^[2]^ Endocrine therapy is the primary treatment modality for hormone receptor-positive breast cancer.^[3]^ Currently, the first-line endocrine therapy for hormone receptor-positive breast cancer involves the use of tamoxifen for premenopausal patients; postmenopausal patients are mainly treated with third-generation aromatase inhibitors, including anastrozole, letrozole, and exemestane.^[4]^ These drugs help control the progression of the disease by regulating hormone levels.

Endocrine therapy is effective for hormone receptor-positive breast cancer but significantly increases the risk of osteoporosis and fractures by lowering estrogen levels in the body. Special attention is needed for hip fractures due to their severity and the associated high mortality rate.^[5,6]^ Estrogen plays a role in inhibiting bone resorption and promoting calcitonin secretion. Most breast cancer patients are in the perimenopausal or postmenopausal phase, during which women’s estrogen levels naturally decline, potentially leading to bone loss and decreased bone density.^[5,7]^ After endocrine therapy, due to the loss of estrogen activity, this bone loss may worsen. Modern Western medicine typically uses a relatively singular approach to prevent bone loss induced by breast cancer endocrine therapy, primarily focusing on bisphosphonate treatment, along with supplements of calcium and vitamin D.^[7]^ Although bisphosphonates can increase bone density, they have no significant effect on alleviating clinical symptoms of bone loss, and their high cost increases the economic burden on patients.^[8]^ Recent studies in traditional Chinese medicine (TCM) have identified a close relationship between bone loss and the kidneys. TCM treatments that nourish the kidneys have shown unique advantages in treating bone loss.^[9]^ Kidney-nourishing formulas not only promote bone formation but also effectively inhibit bone resorption, thereby effectively preventing bone loss.^[9,10]^ Moreover, kidney-nourishing treatments have proven to significantly alleviate the musculoskeletal symptoms related to endocrine therapy in breast cancer patients. Studies have shown that TCM kidney-nourishing treatments can effectively relieve musculoskeletal symptoms, including soreness and pain in the lower back and limbs, that occur during endocrine therapy with aromatase inhibitors.^[11]^

Currently, kidney-nourishing treatments have shown significant effectiveness in clinical settings for bone loss related to endocrine therapy for breast cancer. However, due to the lack of uniformity in sample sizes and variations in study design and efficacy assessment methods across studies, the effectiveness of kidney-nourishing treatments still lacks sufficient evidence-based medical support.^[12]^ To address this gap, this study will collect extensive data from randomized controlled trials on kidney-nourishing treatments for bone loss caused by breast cancer endocrine therapy and apply meta-analysis to provide more rigorous and scientific evidence-based medical evidence to verify the effectiveness of these treatments.

## 2. Data and methods

### 2.1. Inclusion criteria for the study

#### 2.1.1. Population (P)

Patients who have received endocrine therapy for breast cancer.

#### 2.1.2. Intervention (I)

TCM kidney-tonifying methods (either TCM alone or integrated with Western medicine).

#### 2.1.3. Comparison (C)

No intervention or Western medical treatment (such as zoledronic acid, calcium supplements).

#### 2.1.4. Outcomes (O)

Lumbar spine bone density, femoral neck bone density, T-score of lumbar spine bone density, clinical efficacy, and drug safety.

#### 2.1.5. Study design (S)

This study is based on clinical randomized controlled trials on the effects of TCM kidney-tonifying methods on bone loss following endocrine therapy for breast cancer.

### 2.2. Exclusion criteria for the study

Studies that do not include the outcome measures of this research. Non-randomized controlled trials, quasi-randomized controlled trials, or pseudo-randomized controlled trials (such as meta-analyses, reviews, case reports, conference papers). Studies with imprecise original trial designs, inaccessible full texts, or incomplete data. Studies not involving TCM kidney-tonifying methods (either TCM treatment or integrated TCM and Western medicine). Studies rated zero by the Cochrane Handbook.

### 2.3. Literature search strategy

The literature search for this study was conducted by 2 authors (second and third authors) and covered 6 major medical electronic databases, including China National Knowledge Infrastructure (CNKI), Wanfang Data, VIP Database, PubMed, Web of Science, and Cochrane Central Register of Controlled Trials. The search terms used included “kidney-tonifying method,” “TCM” “randomized controlled trial,” “Chinese herbal medicine,” “Chinese patent medicine,” “breast cancer,” “bone metastasis,” “osteoporosis,” and “bone loss.” The search covered the period from the inception of each database until April 2024.

### 2.4. Literature screening and data extraction

In this study, literature screening and exclusion were performed using EndNote bibliography management software. Initially, the second and third authors were responsible for eliminating duplicate studies and selecting preliminary research papers. They then excluded non-randomized controlled trials, quasi-randomized controlled trials, and pseudo-randomized controlled trials (such as systematic reviews, meta-analyses, reviews, case reports, and conference papers) to form a second-stage list of literature. Subsequently, by reading titles and abstracts, they excluded studies unrelated to the treatment of bone metastasis or osteoporosis in breast cancer using TCM kidney-tonifying methods, forming the third stage of literature. Finally, by full-text reading, studies that did not meet the study’s outcome measures, had imprecise original trial designs, inaccessible full texts, incomplete data, or were rated zero by the Cochrane Handbook were excluded. This process was independently carried out by the 2 authors, and any disagreements were discussed and resolved with the first author. Ultimately, an exhaustive characteristics table of included literature was created, detailing the first author, year of publication, participants’ age (mean ± standard deviation), number of participants in the experimental and control groups, intervention measures, control measures, and study outcomes.

### 2.5. Literature quality assessment

This study assessed the quality of RCT literature using the Cochrane Handbook version 5.1.0 ROB tool. A score of 1–3 was considered low quality, while a score of 4 to 7 was considered high quality. The assessment was based on the following 7 aspects: random sequence generation (0–1 points), allocation concealment (0–1 points), blinding of participants and personnel (0–2 points), blinding of outcome assessment (0–2 points), completeness of outcome data (0–1 point), selective reporting (0–1 point), other sources of bias (0–1 point).

### 2.6. Statistical analysis

In this study, to assess the impact of TCM kidney-tonifying methods on bone density and clinical efficacy, appropriate statistical models were selected based on the level of heterogeneity for data synthesis. A random-effects model was used in cases of high heterogeneity, while a fixed-effects model was employed for lower heterogeneity to evaluate the relative risk (RR).^[13]^ The standardized mean difference (SMD) was used to quantify the magnitude of the treatment effect. To verify the robustness of the results, sensitivity analysis was conducted by excluding each study 1 by 1 to observe its impact on the overall effect.^[14]^ Additionally, publication bias was assessed by examining the symmetry of funnel plots to ensure the reliability of the study findings.^[15]^

#### 2.6.1. Ethical approval

This meta-analysis study did not require patient consent because it did not directly involve patients or use their personal information. The data used in the study were sourced from published literature, which are considered reliable and authentic. Therefore, no ethical review and approval were necessary.

## 3. Results

### 3.1. Literature search results

**Figure 1.**
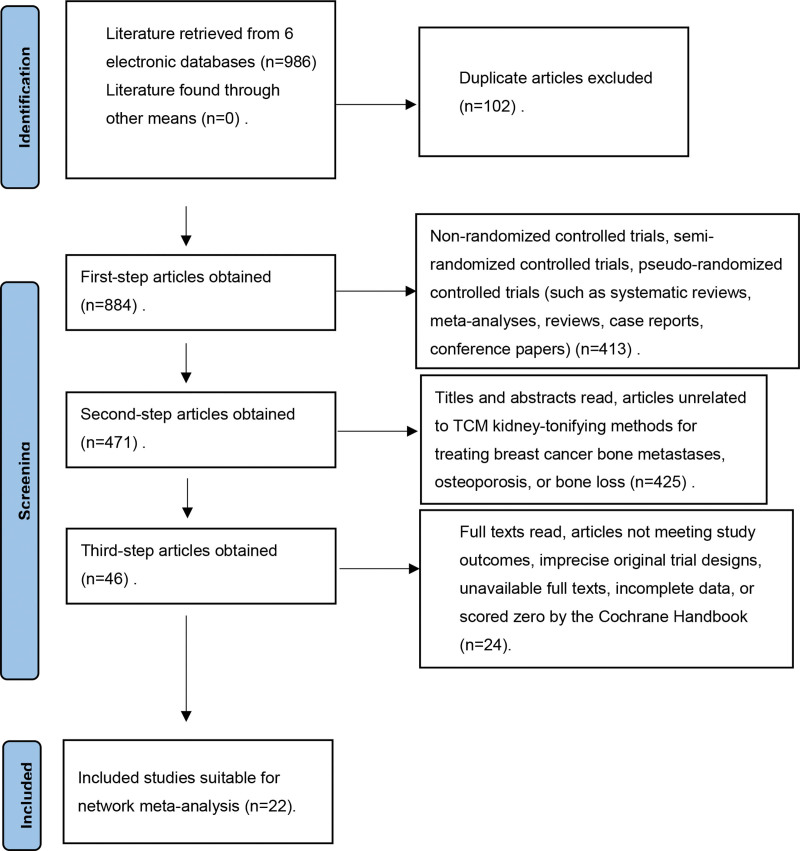
Flowchart of literature screening process.

A search through 6 major medical electronic databases yielded 986 articles, with no additional articles found through other avenues. After excluding 102 duplicate articles, 413 non-randomized controlled trials, semi-randomized controlled trials, and pseudo-randomized controlled trials (such as systematic reviews, meta-analyses, reviews, case reports, and conference papers) were also excluded. By reviewing titles and abstracts, 425 articles unrelated to the treatment of breast cancer bone metastases, osteoporosis, or bone loss with TCM kidney-tonifying methods were removed. The remaining 46 articles were subjected to full-text review, leading to the exclusion of those not meeting the study’s outcome measures, those with imprecise original trial designs, unavailable full texts, incomplete data, or those scored zero by the Cochrane Handbook. Ultimately, 22 articles were included in this study. Details are illustrated in Figure 1.

### 3.2. Summary of the basic characteristics of the included literature

This study included 22 randomized controlled trials with a total of 1579 patients. The analysis of outcome measures involved 12 articles for lumbar spine bone density, 11 articles for lumbar spine bone density *T*-values, 10 articles for femoral neck bone density, 5 articles for clinical efficacy, and 10 articles for drug safety analysis. Details are provided in Table 1.

**Table 1:** Basic characteristics of the included studies in this research.

Author	Year	Age (mean ± SD)	Number of participants (treatment/control)	Intervention	Control measures	Outcome measures
Huang Xiaona^[16]^	2020	C: 51.4 (36–65)	C: 28	Kidney and liver nourishing formula + calcium carbonate D3 tablets	Calcium carbonate D3 tablets	3, 5
		T: 48.3 (34–68)	T: 30			
Liu Hui^[17]^	2019	C: 63.84 ± 7.92	C: 32	Bone density capsules + caltrate D	Caltrate D	1, 4, 5
		T: 63.± 8.15	T: 32			
Liang Lichun^[18]^	2019	C: 62.743 ± 8.027	C: 35	Kidney-tonifying Chinese medicine + caltrate D	Caltrate D	2, 4, 5
		T: 62.257 ± 7.437	T: 35			
Qu Wenchao^[19]^	2019	C: 59. 86 ± 6. 35	C: 64	Spleen and kidney strengthening Chinese medicine	Placebo control	3
		T: 60. 16 ± 5. 68	T: 62			
Sun Zhibo^[20]^	2018	C: 61.95 ± 7.12	C: 50	Kidney and spleen nourishing formula + caltrate D	Caltrate D	4
		T: 61.84 ± 7.73	T: 50			
Zou Suwen^[21]^	2018	C: 57.5 ± 6.1	C: 32	Kidney strengthening and muscle fortifying capsules + caltrate D	Caltrate D	3, 5
		T: 56.2 ± 5.7	T: 33			
Yin Yulian^[22]^	2018	C: 58. 86 ± 7. 047	C: 58	Kidney and bone-strengthening formula + standard Chinese medicine + calcium supplements + vitamin D	Standard Chinese medicine + calcium Supplements + vitamin D	2, 3, 4, 5
		T: 58. 25 ± 5. 973	T: 58			
Zhao Wei^[23]^	2018	C: 56.2 ± 67.0	C: 21	Bone-strengthening and pain relief formula	Caltrate D	1, 2, 4, 5
		T: 58.4 ± 8.9	T: 26			
Li Juanjuan^[24]^	2017	C: 56.1 (50–63)	C: 35	Kidney-tonifying Chinese medicine + caltrate D	Caltrate D	2, 3, 4, 5
		T: 55.6 (49–64)	T: 35			
Shi Hang^[25]^	2017	C: 35.67 ± 10.23	C: 17	Dragon and Ox bone supplement soup + caltrate D	Caltrate D	2, 3, 4
		T: 36.92 ± 9.52	T: 23			
Zhu Qiaoli^[26]^	2017	C: 66.03 ± 5.14	C: 46	Fairy spirit bone preservation capsules + calcium carbonate tablets	Calcium carbonate tablets	2
		T: 67.36 ± 4.67	T: 46			
Bai Jianyun^[27]^	2017	C: 62.8 ± 2.7	C: 45	Kidney benefiting and bone-strengthening soup + calcium carbonate chewable tablets	Calcium carbonate chewable tablets	1, 2, 4, 5
		T: 63.2 ± 3.2	T: 45			
Yuan Bo^[28]^	2016	C: 56.8 ± 6.3	C: 35	Kidney-nourishing and blood-activating formula + caltrate D	Caltrate D	3
		T: 57.9 ± 5.8	T: 35			
Huang Yingfei^[29]^	2016	C: NA (50–65)	C: 30	Kidney strengthening and muscle fortifying capsules + Ba Duan Jin exercises + caltrate D	Caltrate D	3
		T:NA (50–65)	T: 30			
Zhang Liping^[30]^	2016	C: 62 (48–75)	C: 41	Osteoporosis cream + zoledronic acid + calcium supplements	Zoledronic acid + calcium supplements	3
		T: 62 (48–75)	T: 42			
Lu Xiaojun^[31]^	2016	C: 58.34 ± 10. 63	C: 35	Dual yellow bone benefiting formula + caltrate D + calcitriol	Caltrate D + calcitriol	2
		T: 62.71 ± 11.24	T: 35			
Mo Ting^[32]^	2015	C: 63.1 ± 3.4	C: 34	Kidney-tonifying and channel-unblocking formula + calcium carbonate chewable tablets	Calcium carbonate chewable tablets	1, 2, 4
		T: 64.7 ± 4.1	T: 34			
Jiang Shenjun^[33]^	2015	C: 57.9 (53–66)	C: 20	Liver-soothing and kidney-nourishing formula + caltrate D	Caltrate D	1, 5
		T: 58.3 (51–67)	T: 21			
Lu Xiaohao^[34]^	2014	C: 52.3 (50–64)	C: 35	Kidney-nourishing and blood-activating formula + caltrate D	Caltrate D	3
		T: 53.6 (49–61)	T: 39			
Xu Juan^[35]^	2014	C: 55. 1 ± 8. 8	C: 40	Liuwei Dihuang pills + vitamin D calcium preparation	Vitamin D calcium preparation	2
		T: 58. 6 ± 10. 7	T: 32			
Li Yuanqing^[36]^	2014	C: 60 (37–66)	C: 17	Liver-soothing and bone-strengthening formula + Caltrate D	Caltrate D	2, 4, 5
		T: 60 (36–67)	T: 21			
Chen Guifen^[37]^	2013	C: 61.13 ± 11.31	C: 28	Liuwei Dihuang Pills	Placebo control	2, 3
		T: 61.35 ± 10.11	T: 28			

C: control group; T: treatment group; 1: clinical efficacy; 2: lumbar spine bone density; 3: lumbar spine bone density *T*-value; 4: femoral neck bone density; 5: Drug safety, NA: not available.

### 3.3. Results of literature quality assessment

**Figure 2.**
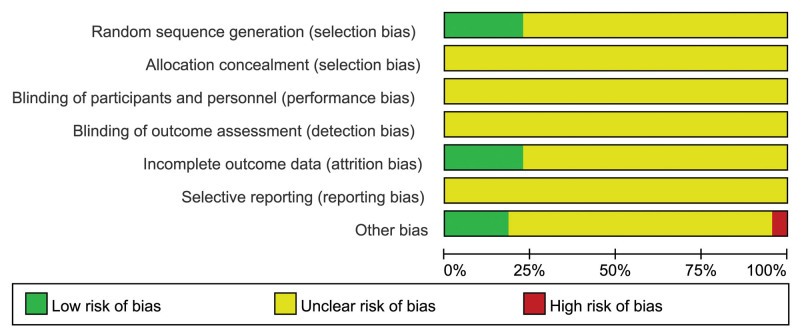
Risk of bias assessment for included studies.

In this study, 3 articles were defined as high-quality literature, while nineteen were classified as low-quality literature. All twenty-two studies included utilized random assignment, with 5 employing a “random number table method” while the remaining studies did not specify their randomization methods in detail. Five studies clearly reported data completeness and selective reporting, with no studies explicitly stating whether they had engaged in selective reporting. Detailed results can be seen in Figure 2 and Table 2.

**Table 2:** Cochrane quality score table for included literature.

Article ID	Article name	Total score	Literature quality
1	Huang Xiaona.2020	1	Low quality
2	Liang Lichun.2019	2	Low quality
3	Liu Hui.2019	2	Low quality
4	Qu Wenchao.2019	4	High quality
5	Sun Zhibo.2018	1	Low quality
6	Yin Yulian.2018	2	Low quality
7	Zhao Wei.2018	3	Low quality
8	Zou Suwen.2018	3	Low quality
9	Bai Jianyun.2017	1	Low quality
10	Li Juanjuan.2017	4	High quality
11	Shi Hang.2017	1	Low quality
12	Zhu Qiaoli.2017	4	High quality
13	Huang Yingfei.2016	1	Low quality
14	Lu Xiaojun.2016	1	Low quality
15	Yuan Bo.2016	1	Low quality
16	Zhang Liping.2016	1	Low quality
17	Jiang Shenjun.2015	1	Low quality
18	Mo Ting.2015	1	Low quality
19	Li Yuanqing.2014	1	Low quality
20	Lu Xiaohao.2014	1	Low quality
21	Xu Juan.2014	1	Low quality
22	Chen Guifen.2013	1	Low quality

### 3.4. Meta-analysis results

#### 3.4.1. Lumbar spine bone density

**Figure 3.**
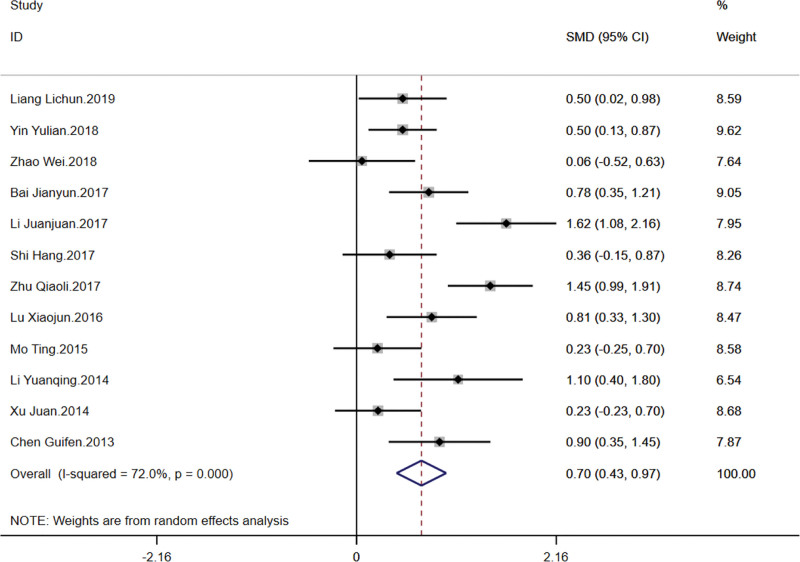
Forest plot for lumbar spine bone density.

This outcome measure included twelve articles involving a total of 848 patients to evaluate the effects of TCM kidney-tonifying methods on lumbar spine bone density through a meta-analysis. The studies exhibited considerable heterogeneity (*I*^2^ = 72%), suggesting that a random-effects model was appropriate for the combined analysis. The overall SMD was 0.70, with a 95% confidence interval (CI) from 0.43 to 0.97, and the *P*-value for heterogeneity was <.001, indicating significant variability among the studies. This demonstrates that TCM kidney-tonifying methods significantly improved lumbar spine bone density compared to control groups. Specific analysis revealed that the study “Li Juanjuan.2017” had a significant contribution to the overall effect, with an SMD of 1.62 and a 95% CI from 1.08 to 2.16, carrying a weight of 7.95%, while “Zhao Wei.2018” reported an SMD of 0.06 with a 95% CI from −0.52 to 0.63, contributing a weight of 7.64%, with a smaller effect size and greater uncertainty. The overall combined effect [SMD = 0.70, 95% CI (0.43, 0.97), *P* < .001] thoroughly demonstrates the efficacy of TCM kidney-tonifying methods in enhancing lumbar spine bone density. The forest plot for this analysis is shown in Figure 3.

**Figure 4.**
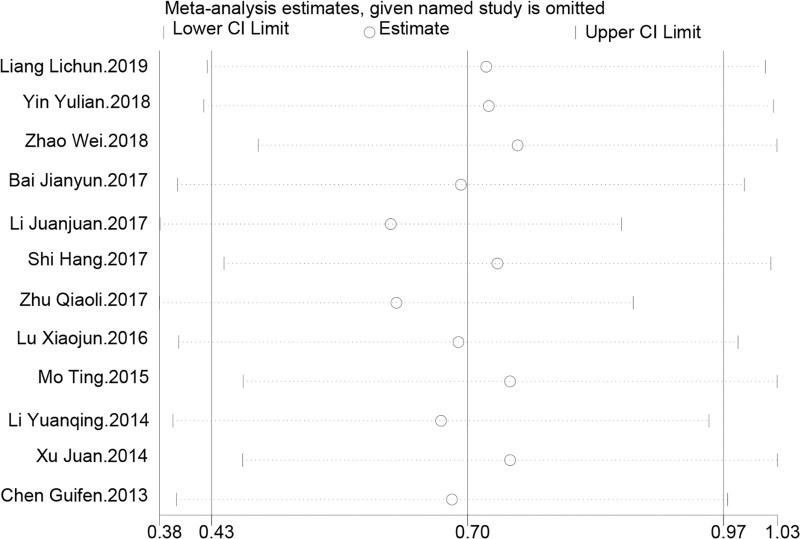
Sensitivity analysis for lumbar spine bone density.

Sensitivity analysis indicated that the meta-analysis results were highly robust to the removal of any single study. With the exclusion of each study, the combined effect estimates for the improvement in lumbar spine bone density remained stable, approximately at an SMD of 0.70, with the 95% CI consistently between 0.38 and 1.03. This suggests that no single study unduly influenced the overall analysis results. The final comprehensive estimate of the SMD was 0.703, with a 95% CI from 0.434 to 0.971, further confirming the significant positive impact of TCM kidney-tonifying methods on lumbar spine bone density, with good consistency across different studies and reliable conclusions as shown in Figure 4.

#### 3.4.2. Lumbar spine bone density *T*-score

**Figure 5.**
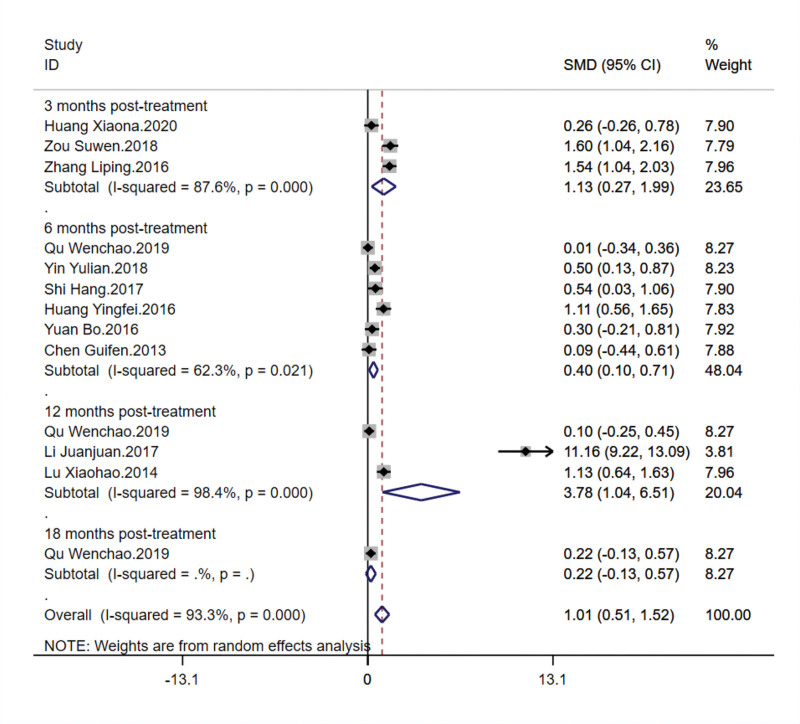
Forest plot for lumbar spine bone density *T*-value.

This outcome measure included eleven articles involving 832 patients to assess the impact of TCM kidney-tonifying methods on the lumbar spine bone density *T*-score. Significant heterogeneity among studies was observed at different time points posttreatment, with *I*^2^ values of 87.6% at 3 months, 62.3% at 6 months, and 98.4% at 12 months, and a total *I*^2^ of 93.3%. A random-effects model was therefore chosen for the overall analysis. At 3 months posttreatment, the combined SMD was 1.129, with a 95% CI from 0.272 to 1.987, *P* < .001. At 6 months, the combined SMD was 0.405, with a 95% CI from 0.100 to 0.709, *P* = .021. At 12 months, despite some extreme values reported in individual studies, the combined SMD was 3.778, with a 95% CI from 1.044 to 6.512, *P* < .001. At 18 months, only 1 study contributed an SMD of 0.216, with a 95% CI from −0.134 to 0.567, and the *P*-value was 0.226, showing no statistical significance. The overall analysis results [SMD = 1.012, 95% CI (0.506, 1.517), *P* < .001] confirmed that TCM kidney-tonifying methods significantly improved the lumbar spine bone density *T*-score. Although there was considerable variation in effects across different time points, overall, TCM kidney-tonifying methods significantly improved the lumbar spine bone density *T*-score. Forest plots are shown in Figure 5.

**Figure 6.**
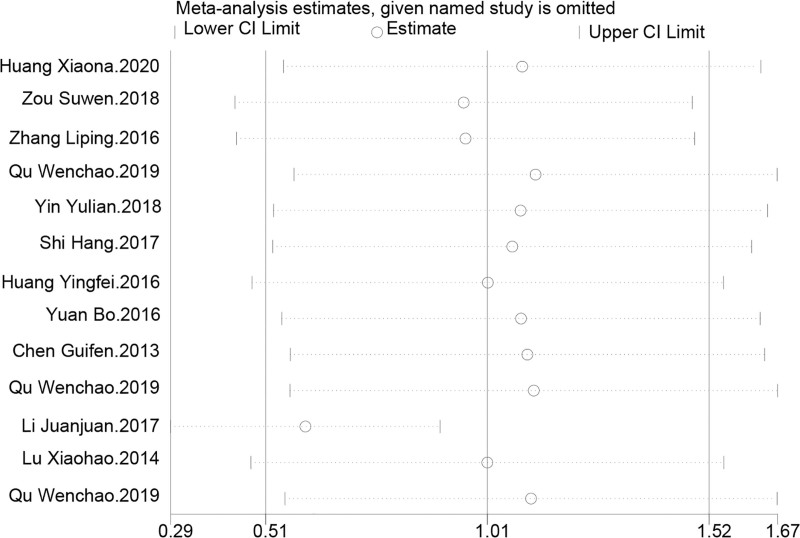
Sensitivity analysis for lumbar spine bone density *T*-value.

Sensitivity analysis revealed that even with the exclusion of different studies 1 at a time, the combined effect estimates and their 95% CIs for the impact of TCM kidney-tonifying methods on the lumbar spine bone density T-score changed little, remaining around 1.01, indicating good stability of results. The exclusion of the “Li Juanjuan.2017” study significantly changed the effect estimate (0.597), prompting a reevaluation of the article, which revealed a smaller sample size and a different herbal composition and dosage compared to other studies. The exclusion of other studies did not cause the effect estimates or CIs to cross the line of no effect. The overall combined effect estimate was 1.012, with a 95% CI from 0.506 to 1.517, further confirming the consistent improvement effect of TCM kidney-tonifying methods on lumbar spine bone density *T*-values under the influence of different studies. Therefore, it can be concluded that TCM kidney-tonifying methods have a positive impact on the lumbar spine bone density *T*-value, and the variability of individual study results does not affect this overall trend, as shown in Figure 6.

### 3.5. Femoral neck bone density

**Figure 7.**
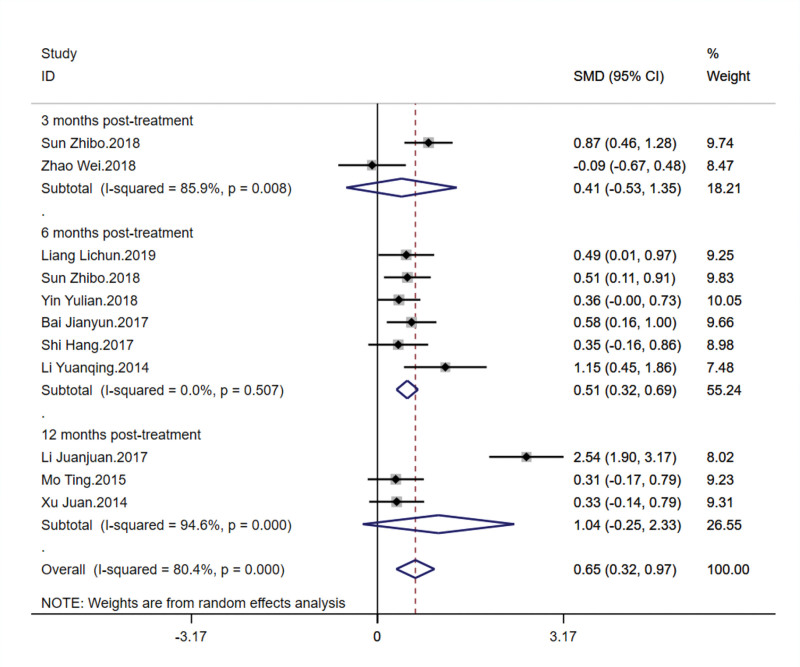
Forest plot for femoral neck bone density.

This outcome included ten studies with a total of 730 patients, assessing the effect of TCM kidney-tonifying methods on femoral neck bone density. Significant heterogeneity was observed among the studies at different time points posttreatment: *I*^2^ = 85.9% at 3 months, *I*^2^ = 0.0% at 6 months, and *I*^2^ = 94.6% at 12 months, with overall heterogeneity at 80.4%. A random-effects model was used for analysis. At 3 months, the combined SMD was 0.410 with a 95% CI from −0.530 to 1.350, not statistically significant (*P* = .393). At 6 months, the combined SMD was 0.507 with a 95% CI from 0.323 to 0.691, statistically significant (*P* < .001), indicating that TCM kidney-tonifying methods significantly improved femoral neck bone density at this time point. At 12 months, although individual study reports showed extreme values, the combined SMD was 1.040 with a 95% CI from −0.249 to 2.330, not statistically significant (*P* = .114). Overall, TCM kidney-tonifying methods significantly improved femoral neck bone density [SMD = 0.645, 95% CI (0.321, 0.970), *P* < .001]. These results suggest that despite significant variability between studies, TCM kidney-tonifying methods generally have a positive effect on femoral neck bone density, particularly noticeable at 6 months posttreatment. Forest plots are displayed in Figure 7.

**Figure 8.**
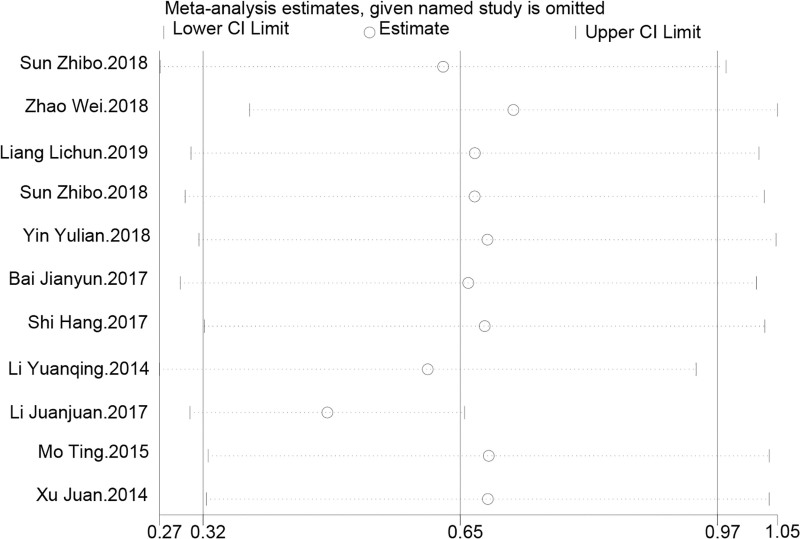
Sensitivity analysis for femoral neck bone density.

Sensitivity analysis revealed that even with the exclusion of individual studies, the combined effect estimates and their 95% CIs for the impact of TCM kidney-tonifying methods on femoral neck bone density remained stable. The overall effect estimate was 0.645, with a CI ranging from 0.321 to 0.970, demonstrating good consistency. Apart from the removal of the “Li Juanjuan.2017” study, which significantly lowered the estimate, the exclusion of other studies did not cause significant changes to the effect values or CIs. This is similar to the results for lumbar spine bone density *T*-values, strengthening the conclusion that TCM kidney-tonifying methods may have a positive effect on increasing femoral neck bone density. This conclusion remains robust despite the variability across different studies, as shown in Figure 8.

### 3.6. Clinical efficacy

**Figure 9.**
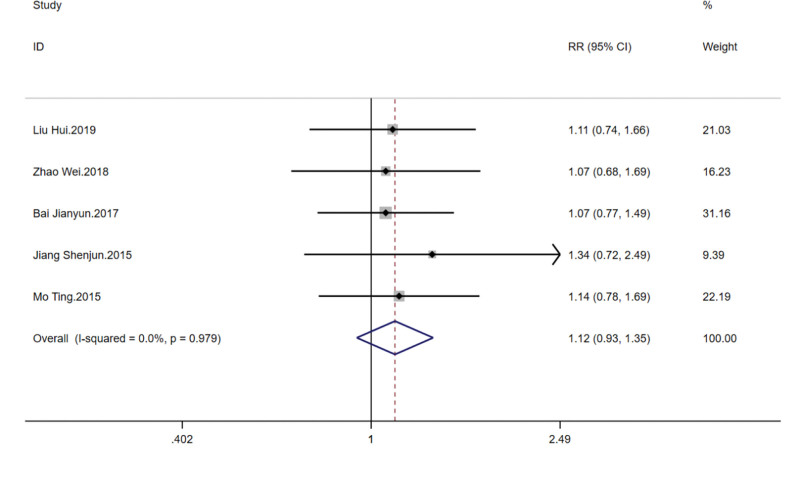
Forest plot for clinical efficacy.

This outcome involved 5 studies covering 310 patients to assess the impact of TCM kidney-tonifying methods on clinical efficacy. The heterogeneity among the included studies was extremely low (*I*^2^ = 0.0%, *P* = .979), thus a fixed-effects model was used for analysis. The summarized RR was 1.122 with a 95% CI from 0.933 to 1.348, indicating a trend towards improvement in clinical efficacy with TCM kidney-tonifying methods, though not reaching statistical significance (*z* = 1.22, *P* = .222). This may suggest that TCM kidney-tonifying methods positively influence treatment outcomes, but due to limited sample sizes or the modest magnitude of effect, further validation in larger-scale studies is necessary. Forest plots are shown in Figure 9.

**Figure 10.**
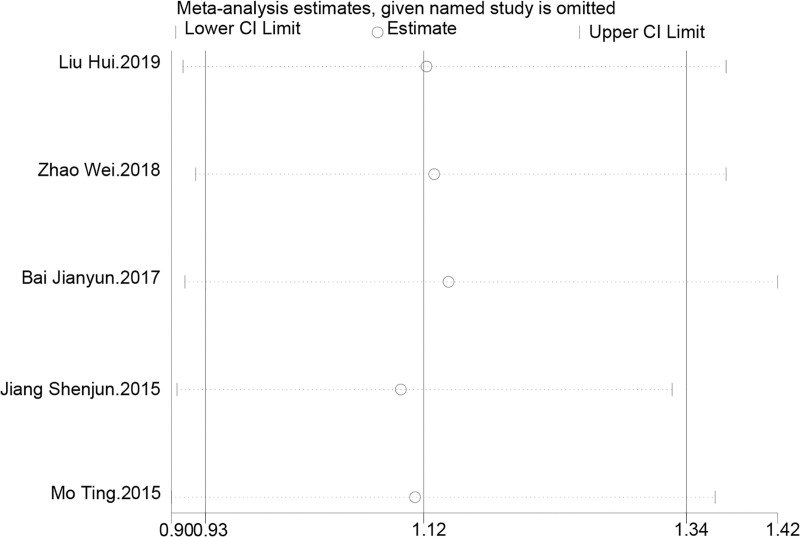
Sensitivity analysis for clinical efficacy.

Further sensitivity analysis indicated that after the exclusion of any single study from the meta-analysis, the estimated RR and their 95% CIs displayed good consistency. When individual studies were sequentially omitted, the estimated RRs fluctuated slightly but remained between 1.09 and 1.14, indicating that each study contributed evenly to the overall result without any single study having an undue influence. The combined RR was 1.118, with a 95% CI from 0.930 to 1.344, reaffirming the consistent positive impact of the assessed TCM kidney-tonifying methods on improving clinical efficacy, with low sensitivity to individual studies, as shown in Figure 10.

### 3.7. Drug safety

This outcome measure included ten studies^[16–18,21–24,27,33,36]^ analyzing drug safety, with 8 articles reporting no significant adverse reactions. Only 2 papers^[24,36]^ reported a case of lower limb fractures in the control group each. These results further confirm the safety of the drugs used in TCM kidney-tonifying methods.

### 3.8. Analysis of publication bias

**Figure 11.**
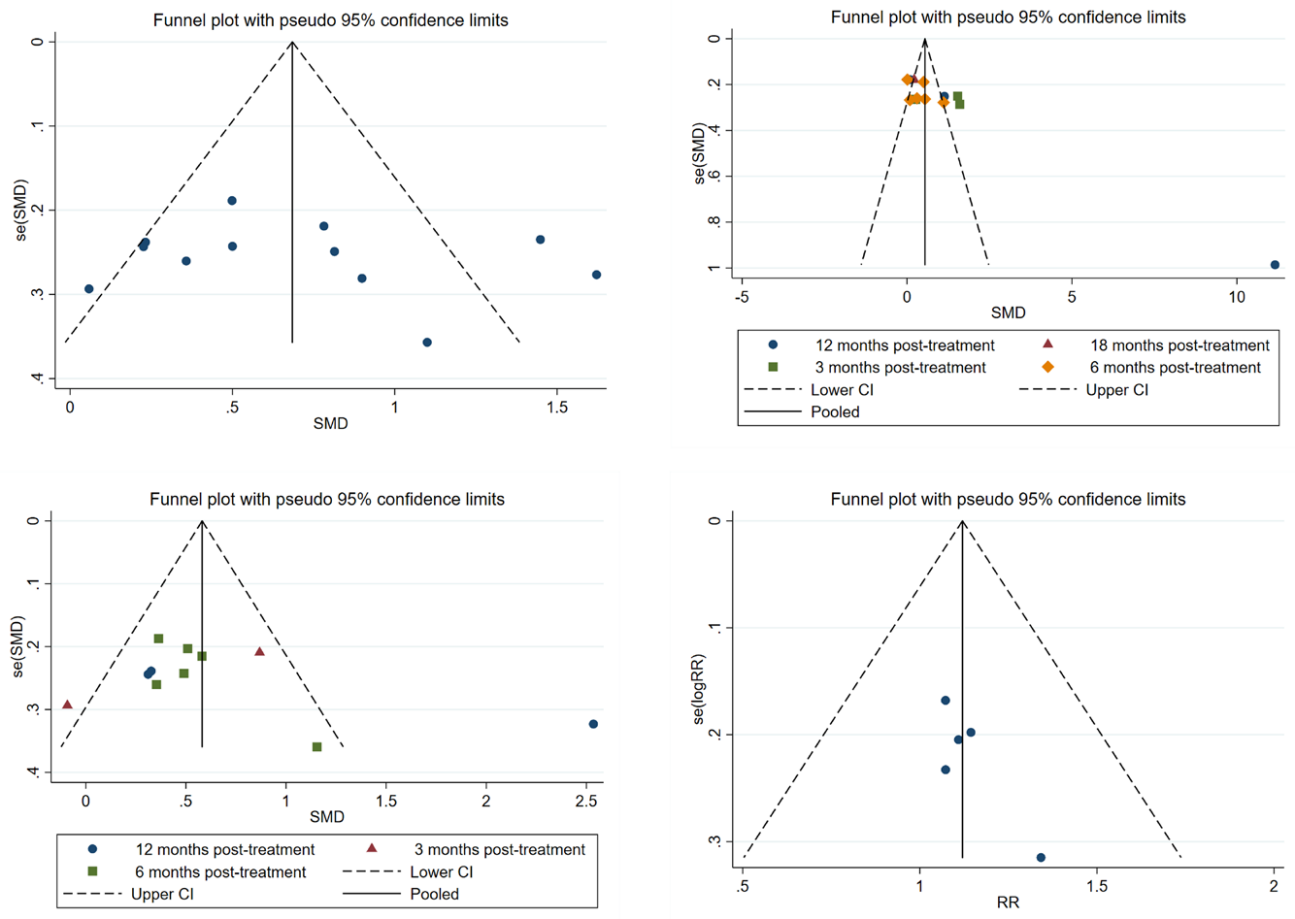
Publication bias analysis of included studies.

The analysis of 4 funnel plots showed good symmetry for lumbar spine bone density and clinical efficacy, suggesting a low risk of publication bias for these outcomes. The funnel plots for femoral neck bone density and lumbar spine bone density *T*-values displayed some asymmetry, possibly due to the influence of the literature pointed out in the sensitivity analysis, but overall, the publication bias was minimal (see Figure 11).

## 4. Discussion

This meta-analysis, incorporating 22 randomized controlled trials involving 1579 patients, comprehensively assessed the impact of TCM kidney-tonifying methods on bone density and clinical efficacy. The results indicate that TCM kidney-tonifying methods significantly increased lumbar spine bone density (SMD = 0.70, 95% CI: 0.43–0.97, *P* < .001) and lumbar spine bone density *T*-values (SMD = 1.012, 95% CI: 0.506–1.517, *P* < .001), showing significant efficacy in enhancing bone density. Additionally, although the improvement in femoral neck bone density did not reach the same level of statistical significance as the lumbar spine, it also demonstrated a positive trend (SMD = 0.645, 95% CI: 0.321–0.970, *P* < .001). Despite the improvements in clinical efficacy not being significant (RR = 1.122, 95% CI: 0.933–1.348, *P* = .222), the overall study results support the applicability of TCM kidney-tonifying methods in enhancing bone density, offering important guidance for clinical practice.

In hormone receptor-positive breast cancer patients, bone loss following endocrine therapy increases the risk of osteoporosis and fractures. Typical symptoms of osteoporosis include bone pain, muscle weakness, reduced height, and potentially severe outcomes such as spinal deformity and fractures.^[5,38]^ Therefore, bone loss posttreatment is a significant clinical issue that requires attention and preventative measures. In TCM, conditions related to bone loss, osteoporosis, and fractures are categorized under the “bone bi” syndrome.^[9]^ Historical Chinese medical practitioners often approached and treated bone loss from the perspective of kidney nourishment, attributing it to essential weakness (ben deficiency) and manifesting as excessive symptoms (biao excess).^[39]^ Ancient texts, such as “Su Wen·Yin Yang Ying Xiang Da Lun,” explicitly state, “The kidneys generate bone marrow, and when kidney essence is sufficient, bone marrow is abundantly produced,” highlighting the direct link between kidney essence and bone health. When kidney essence is abundant, bone marrow is sufficiently generated, maintaining bone strength and vitality; conversely, insufficient kidney essence can lead to osteoporosis, resulting in fragile and weak bones that lack resilience. Recent studies, such as 1 by Lu et al,^[40]^ have shown that using Guigu capsules (kidney-nourishing) to treat osteoporosis significantly improves bone mineral density and serum markers of bone metabolism, indicating potential benefits for treatment and prevention. A recent systematic review^[41]^ also suggested that combining TCM with Western medicine could offer more gentle and personalized treatment options for osteoporosis by enhancing hormones like estrogen that are beneficial for bone formation and thus improving bone density.

The positive results on bone density in this study are supported by previous literature,^[42,43]^ including a 5-year multicenter follow-up study^[43]^ that observed the preventive effects of a kidney-nourishing herbal formula on osteoporosis and fractures in postmenopausal women. The results showed that the group using the kidney-nourishing herbal formula had significantly increased bone density and a lower rate of fractures compared to controls, demonstrating the potential of kidney-nourishing herbs to prevent postmenopausal osteoporosis and reduce the risk of brittle fractures. Another study^[42]^ assessed the long-term impact of a kidney-nourishing formula on improving bone density in kidney transplant patients. The results indicated that patients using the kidney-nourishing formula had significantly higher bone density post-transplant and reduced fracture risk, proving the effectiveness of kidney-nourishing medications in long-term management of osteoporosis and fracture reduction. Therefore, it is not surprising that TCM kidney-nourishing methods significantly enhanced lumbar spine bone density, lumbar spine bone density *T*-values, and femoral neck bone density in this study, showing significant efficacy in enhancing bone density. However, the improvement in clinical efficacy was not significant (RR = 1.122, 95% CI: 0.933–1.348, *P* = .222), which is consistent with previous literature. One study^[44]^ compared traditional TCM kidney-nourishing methods with modern medical treatments for osteoporosis. While the kidney-nourishing methods improved bone density, the effects were not significant in some cases, necessitating more clinical data to support their widespread application. A recent systematic review and meta-analysis^[45]^ investigated the effectiveness of TCM kidney-nourishing principles in treating primary osteoporosis. Although some studies showed good results in enhancing bone density when kidney-nourishing methods were used in conjunction with conventional Western medications, overall evidence for their safety and efficacy remains insufficient.

This study has some limitations. Firstly, most included studies are of low quality, potentially affecting the reliability of results. Additionally, high heterogeneity among studies, especially observed at different time points for lumbar spine bone density *T*-values, suggests inconsistencies in underlying research design and implementation. Individual studies like “Li Juanjuan.2017” may have significantly influenced overall effect assessments due to small sample sizes and different treatment dosages. Although some indicators like lumbar spine bone density and *T*-values showed statistical improvements, the lack of significant improvement in clinical efficacy (RR = 1.122, 95% CI: 0.933–1.348, *P* = .222) indicates that the effectiveness of TCM kidney-nourishing methods in clinical practice may be limited. Lastly, although some results’ funnel plots show good symmetry, the risk of publication bias should still be cautiously considered. These limitations could affect the generalizability and applicability of the study’s conclusions.

In summary, the meta-analysis results of this study demonstrate that TCM kidney-nourishing methods significantly enhance lumbar spine bone density, lumbar spine bone density *T*-values, and femoral neck bone density, showing their effectiveness in enhancing bone density. Although results for improving clinical efficacy did not reach statistical significance, the study indicates that the therapy has good safety, with most studies reporting no significant adverse effects. These findings support the potential use of TCM kidney-nourishing methods as a preventive and therapeutic approach for osteoporosis and other bone-related diseases, providing important insights for future clinical applications.

## Author contributions

**Data curation:** Yuanqi Zhang, Jianwen Li.

**Writing – original draft:** Liuxiang Chen.

**Writing – review & editing:** Jianwen Li, Attila Kalmar.
